# Loneliness, problematic digital media use, and nutritional–metabolic vulnerability in adolescents from single-parent households: an integrative review of eating- and sleep-related pathways

**DOI:** 10.3389/fnut.2026.1888958

**Published:** 2026-09-22

**Authors:** Xuemei Zhang

**Affiliations:** School of Marxism, Shaanxi University and Technology, Hanzhong, Shaanxi, China

**Keywords:** emotional eating, loneliness, metabolic risk, nutritional vulnerability, problematic internet use, single-parent adolescents, sleep disruption, weight-related outcomes

## Abstract

Adolescents from single-parent households may experience household time, economic, supervisory, and routine-related constraints that are relevant to psychosocial well-being, digital media use, eating behavior, sleep, and metabolic health. The objective of this integrative review was to synthesize evidence on the associations of loneliness and problematic digital media use with eating- and sleep-related behaviors in adolescents and to examine how single-parent household characteristics may influence these relationships. The review applies a contextual–behavioral vulnerability model in which household characteristics are treated as contextual modifiers; loneliness and problematic digital media use as the principal psychosocial and behavioral exposures; emotional eating, irregular meal timing, distracted eating, sleep disruption, circadian delay, and sedentary behavior as candidate intermediate processes; and diet quality, adiposity, and metabolic vulnerability as distal outcomes. Eligible evidence included peer-reviewed empirical studies, systematic reviews, and meta-analyses focused primarily on adolescents and examining at least one relevant association among family structure, loneliness, digital media use, eating behavior, sleep, energy balance, adiposity, or metabolic indicators. Studies explicitly analyzing single-parent households were treated as direct evidence, whereas broader adolescent or mechanistic studies were included when direct evidence was limited and were interpreted as indirect evidence. Direct single-parent evidence was concentrated mainly on family meals, screen-related routines, physical activity, and weight-related outcomes. Broader adolescent evidence associated loneliness with emotional eating and problematic digital media use with breakfast skipping, irregular meals, distracted eating, poorer diet quality, and sleep disruption. Evidence linking these behavioral patterns to adiposity, insulin sensitivity, circadian regulation, stress responsivity, and appetite-related signaling remained predominantly indirect and observational. Longitudinal, objectively measured, and biomarker-informed studies are needed to test these relationships directly in adolescents from single-parent households.

## 1 Introduction

Adolescence is a critical developmental period during which dietary habits, physical activity, sleep routines, and patterns of digital-media use become increasingly established. These behaviors have important implications for psychosocial wellbeing, body-weight regulation, and later metabolic health. Family context can influence their organization by shaping the availability of parental time, economic resources, supervision, shared meals, transportation, and access to structured activities. Single-parent households are therefore relevant not because family structure independently determines poor health, but because time, financial, and organizational constraints may converge in some families. Studies have reported less frequent family meals, lower participation in organized physical activity, greater screen-based sedentary behavior, and less favorable weight-related outcomes in some single-parent samples, although findings vary across populations and socioeconomic settings (1–3).

Digital media constitute an additional influence on adolescent routines. Screen exposure may displace physical activity, structured meals, and sleep, while food-related content, advertising, gaming, and social-media engagement may coincide with snacking, breakfast omission, and less favorable dietary choices. Screen time refers to the duration or frequency of engagement with screen-based devices or activities, irrespective of whether control is impaired. Problematic digital media use refers to a pattern characterized by impaired control, preoccupation, difficulty reducing use, or interference with daily functioning. Accordingly, high screen time should not be interpreted as evidence of problematic use, and problematic use may have different behavioral implications from frequent but controlled engagement. Late-night or compulsive digital engagement has also been associated with delayed bedtime, shorter sleep duration, and reduced sleep quality, although most evidence is observational and does not establish whether digital use precedes these behaviors or emerges in response to existing distress or disrupted routines (4–6).

Psychosocial factors may further interact with these behavioral patterns. Loneliness and perceived social isolation have been associated with emotional or stress-related eating, particularly when negative affect, limited social support, and difficulties in emotion regulation coexist (7). Sleep disruption, reduced physical activity, irregular eating, and prolonged digital engagement may also cluster within the same adolescents, potentially increasing vulnerability to adiposity and cardiometabolic risk without forming a single or predetermined causal sequence (8–10). Examining family context, loneliness, digital-media use, eating behavior, and sleep within an integrated framework may therefore help distinguish contextual vulnerability from behavioral pathways and distal metabolic outcomes.

Although previous studies have examined family structure, loneliness, digital media use, sleep, and eating behavior separately, the conceptual roles of these factors within adolescent nutritional and metabolic vulnerability remain insufficiently differentiated. In particular, single-parent household status is frequently treated as an independent predictor even though the relevant behavioral constraints may arise from more specific household characteristics, including limited parental time, financial pressure, reduced evening supervision, fewer shared meals, greater adolescent household responsibilities, and less stable daily routines. Evidence concerning loneliness, problematic digital media use, sleep disruption, and eating behavior is also derived predominantly from observational studies conducted in general adolescent populations. Accordingly, the evidence base prioritizes peer-reviewed adolescent studies and explicit single-parent analyses, while broader adolescent or mechanistic evidence is considered only when direct evidence is limited and is identified as indirect. These factors should therefore not be organized into a predetermined causal sequence.

This integrative review therefore adopts a contextual–behavioral vulnerability framework. Single-parent household characteristics are considered contextual modifiers that may alter exposure to psychosocial stressors and the organization of daily routines. Loneliness and problematic digital media use are treated as the principal psychosocial and behavioral exposures, whereas emotional eating, irregular meal timing, distracted eating, sleep disruption, circadian delay, and sedentary behavior are evaluated as candidate intermediate processes. Diet quality, energy-related eating behaviors, adiposity, and metabolic vulnerability are considered progressively more distal outcomes. The primary objective is to synthesize evidence on how loneliness and problematic digital media use are associated with eating- and sleep-related behaviors in adolescents and to assess whether these interrelated behaviors may contribute to nutritional and metabolic vulnerability. A secondary objective is to examine how characteristics associated with single-parent households may plausibly modify these relationships. Throughout the review, evidence is classified as direct when studies explicitly analyze single-parent households and as indirect when findings from broader adolescent samples are used to assess the contextual plausibility of the proposed relationships. Because much of the available evidence is observational, the proposed relationships are interpreted as associative, potentially bidirectional, and context-dependent rather than as an established causal pathway. To maintain a nutrition-centered scope, psychological, digital, sleep-related, activity-related, and metabolic evidence is considered only where it directly informs eating behavior, diet quality, adiposity, or nutritional–metabolic vulnerability.

## 2 Methods

This integrative review was conducted as a structured narrative synthesis rather than as a systematic review or meta-analysis. A targeted multi-database literature search was performed in PubMed/MEDLINE, Scopus, Web of Science Core Collection, and APA PsycINFO from database inception to July 20, 2026. Google Scholar, reference lists of relevant reviews and eligible publications, and forward citation tracking were used as supplementary sources. The search was organized around four complementary concept groups: single-parent household characteristics and adolescent health behaviors; loneliness or social isolation and eating- or weight-related outcomes; problematic internet, smartphone, social media, or screen use and diet, eating behavior, sleep, or sedentary behavior; and sleep or circadian disruption and appetite-related, adiposity, insulin-related, or cardiometabolic outcomes. Search terms included combinations of *adolescent*, *teen*, *youth*, *single parent*, *single mother*, *single father*, *family structure*, *loneliness*, *social isolation*, *problematic internet use*, *problematic smartphone use*, *social media use*, *screen time*, *digital media*, *emotional eating*, *diet*, *nutrition*, *breakfast*, *snacking*, *meal timing*, *sleep*, *circadian*, *sedentary behavior*, *physical activity*, *obesity*, *adiposity*, *metabolic risk*, *insulin*, *glucose*, *cortisol*, *leptin*, and *ghrelin*. Search terms were combined using Boolean operators and adapted to the indexing system and search fields of each database. The searches were conducted in titles, abstracts, keywords, and subject headings, where available. Reference lists of relevant reviews and eligible studies were also screened to identify additional publications that may not have been retrieved through the database searches.

Peer-reviewed human studies published in English were eligible when they primarily included adolescents aged 10–19 years, reported separately extractable adolescent findings, or followed participants from adolescence into young adulthood. Eligible publications examined at least one relevant contextual or psychosocial exposure, including single-parent household characteristics, loneliness, social isolation, problematic digital media use, or screen exposure, together with at least one eating-related, sleep-related, activity-related, anthropometric, or metabolic outcome. Primary cross-sectional, longitudinal, qualitative, mixed-methods, and intervention studies were considered. Systematic, scoping, and narrative reviews were used to identify additional primary studies, assess consistency across the literature, or support contextual interpretation, but were not treated as equivalent to primary evidence. Studies explicitly analyzing single-parent, single-mother, or single-father households were classified as direct evidence for the target context. Findings from broader adolescent populations were classified as indirect evidence when used to assess the contextual plausibility of the proposed pathways. Adult-only studies were excluded from the core evidence synthesis, although selected mechanistic publications were retained when adolescent evidence was unavailable and were explicitly interpreted as indirect support.

Non-human studies, case reports, conference abstracts, dissertations, editorials, commentaries, non-peer-reviewed publications, studies without relevant exposures or outcomes, and reports in which adolescent findings could not be separated from adult data were excluded. Publications focused predominantly on academic procrastination or academic performance without direct assessment of eating behavior, sleep, energy balance, adiposity, or metabolic outcomes were also excluded. When multiple publications used the same cohort, each was retained only when it reported a distinct exposure, outcome, follow-up period, or analytical approach relevant to the review.

Duplicate records were removed before titles and abstracts were screened against the predefined eligibility criteria. Potentially relevant publications underwent full-text assessment, and eligibility decisions were recorded in a standardized evidence-extraction file. To reduce inconsistency associated with single-author screening, all included publications and full-text exclusions were re-evaluated in a second screening pass before the synthesis was finalized. For each publication, data were extracted on author and year, country, sample characteristics, age range, study design, sample size, exposure and outcome measures, family-structure analysis, principal findings, covariate adjustment, follow-up duration, and major methodological limitations. Digital-media exposures were coded separately as duration-based screen time, content- or timing-specific exposure, or problematic/compulsive use according to the definitions and measurement instruments used in each study. Findings from these exposure categories were not treated as interchangeable. Each publication was also classified as providing direct, indirect, or mixed evidence for the single-parent context. Methodological strength was appraised qualitatively according to study design, sampling, measurement quality, adjustment for confounding, directness to the target population, consistency with related findings, precision, and capacity to establish temporal ordering. Each body of evidence was classified as limited, limited-to-moderate, or moderate for the specific inference being evaluated. These ratings were inference-specific rather than based on a rigid design hierarchy. Systematic reviews and meta-analyses were not automatically classified as strong evidence because their contribution depended on the design and quality of the included studies, between-study heterogeneity, and the outcomes assessed. Completed randomized controlled trials were considered the most informative designs for intervention effects, whereas trial protocols were not treated as evidence of effectiveness. Prospective cohort studies contributed to temporal ordering but remained susceptible to attrition, residual confounding, and measurement error. Cross-sectional studies supported the presence of associations but not their direction, while qualitative studies informed contextual interpretation rather than effect magnitude. Mechanistic, adult, or non-target-population studies were classified as indirect evidence. No numerical quality score or formal risk-of-bias exclusion threshold was applied because the review was designed to integrate heterogeneous evidence rather than estimate pooled effects.

Longitudinal studies were additionally evaluated according to follow-up duration, number of assessment waves, baseline adjustment, attrition, measurement quality, and control of potential confounding. Temporal inference was described as limited, moderate, or comparatively stronger. Limited inference generally referred to short or two-wave observational studies with important measurement or confounding constraints; moderate inference required clearly separated exposure and outcome assessments with appropriate covariate or baseline adjustment; and comparatively stronger inference was reserved for multiwave studies capable of examining within-person or bidirectional change. These categories indicate the relative strength of temporal ordering and do not establish causality. Analyses using data from named longitudinal cohorts were classified as cross-sectional when exposure and outcome were evaluated at the same assessment point. The follow-up duration, longitudinal design, temporal-inference grade, and principal methodological limitations of the longitudinal studies are summarized in Supplementary Table S1. The qualitative strength, directness, and principal risk-of-bias concerns for each evidence domain are summarized in Supplementary Table S2.

Because the included literature was heterogeneous in population, exposure definition, measurement method, design, follow-up duration, and outcome assessment, statistical pooling was neither intended nor considered appropriate. Evidence was synthesized narratively according to the contextual–behavioral vulnerability framework, with emphasis on the distinction between direct single-parent evidence and contextual extrapolation, the consistency and methodological strength of associations, and the degree to which study design supported temporal interpretation. The synthesis prioritized nutrition-related outcomes, while evidence on sleep, physical activity, psychosocial processes, and biological mechanisms was retained only when directly relevant to eating behavior, diet quality, adiposity, or metabolic vulnerability. Representative studies across the principal evidence domains, including their designs, main findings, methodological limitations, and directness to the target context, are summarized in Table 1.

**Table 1:** Representative studies informing the principal evidence domains of the review.

Evidence domain	Population and design	Main finding	Key limitation	Directness and evidence strength	Representative study
Single-parent context and daily routines	Adolescent cross-sectional study	Single-parent or reconstituted household residence was associated with differences in organized activity and weekday screen exposure	Cross-sectional design, self-reported behavior, and possible socioeconomic confounding	Direct evidence; limited-to-moderate for behavioral associations	(2)
Single-parent context, screen access, and family meals	Cross-sectional adolescent study	Single-mother households showed greater bedroom television access and fewer family meals	Temporal ordering could not be established and household resources may confound the associations	Direct evidence; limited for directionality	(14)
Family structure and screen exposure	Cross-sectional adolescent study	Screen exposure did not differ significantly according to family structure	Context-specific findings and limited generalizability	Direct evidence demonstrating heterogeneity	(3)
Loneliness and eating behavior	Observational adolescent study	Loneliness was associated with emotional or disordered eating-related outcomes	Observational design and limited ability to establish mediation or directionality	Mostly indirect evidence; limited-to-moderate	(25)
Digital media use and dietary behavior	Cross-sectional adolescent study	Problematic digital engagement was associated with less favorable dietary behaviors	Self-reported exposure and diet; reverse causation remains possible	Indirect evidence; limited for causality	(37)
Screen exposure and later diet quality	One-year prospective ABCD analysis	Baseline screen exposure showed small associations with subsequent diet-quality outcomes	Small effect sizes, residual confounding, and relatively short follow-up	Indirect prospective evidence; limited-to-moderate temporal support	(45)
Problematic internet use and sleep	Cross-sectional adolescent mediation analysis	Problematic internet use was associated with poorer sleep quality, with anxiety identified as a statistical mediator	Cross-sectional mediation does not demonstrate a sequential mechanism	Indirect evidence; limited for mediation	(55)
Sleep, screen-related sedentary behavior, and overweight	Cross-sectional adolescent study	Insufficient sleep and high screen-based sedentary time were associated with overweight, with the highest odds observed when both were present	Energy intake and expenditure were not measured; additive and causal effects cannot be inferred	Indirect evidence; limited	(63)
Problematic internet use and weight status	Cross-sectional adolescent study	Problematic internet use was associated with higher odds of overweight/obesity after adjustment for confounders	Residual confounding, self-report, and absence of direct energy-balance measurement	Indirect evidence; limited for causal inference	(69)
Intervention evidence	Review of school breakfast programs	School breakfast programs improved access and participation, while effects on broader outcomes were inconsistent	Heterogeneity of interventions and limited evidence for long-term metabolic effects	Indirect intervention evidence; limited-to-moderate for proximal outcomes	(89)

The table presents representative rather than exhaustive evidence. Evidence-strength ratings are inference-specific and reflect study design, directness to adolescents from single-parent households, measurement quality, confounding control, and capacity to support temporal interpretation. Observed associations do not establish mediation or causality.

## 3 Direct evidence on behavioral routines in single-parent households

Direct evidence concerning single-parent households is concentrated on family-meal patterns, dietary behavior, screen exposure, physical activity, and weight-related outcomes. These studies characterize observed behavioral differences across household structures but do not establish the household mechanisms responsible for those differences. In a systematic review of 10 studies, children from single-parent families showed higher body mass index (BMI) and more obesogenic behaviors than those from dual-parent households, although associations were stronger in girls and selected demographic groups and were not consistent across all populations. These findings indicate that socioeconomic and demographic conditions shape the relationship between family structure and behavioral vulnerability (1, 11).

Evidence from individual studies varies substantially in design and inferential strength. Byrne et al. (12) analyzed data from the Longitudinal Study of Australian Children using cross-sectional models; therefore, despite the longitudinal source cohort, the reported associations provide no evidence of temporal ordering. Girls from single-parent households had higher BMI, and children in these households showed less favorable dietary intake and greater television exposure. Augustine and Kimbro (13), by contrast, used family-structure histories from approximately 9 months of age to preschool age, representing about 3 years of follow-up. Selected unstable cohabiting and single-parent trajectories were associated with a greater likelihood of obesity at preschool age. This repeated-measures design provides limited-to-moderate temporal evidence regarding early-childhood family instability, but its observational nature and preschool population limit direct application to adolescents.

The influence of single-parent family structure extends beyond dietary behaviors, encompassing physical activity and engagement in organized sports. Langøy et al. (2) investigated Norwegian adolescents and found that youth living with a single parent or in reconstituted families engaged in fewer days per week of moderate-to-vigorous physical activity (MVPA) and had lower participation in organized sports compared to peers in dual-parent households. Economic resources, parental time, and logistical support were identified as potential mediating factors, as these influence the ability of parents to facilitate structured physical activity, provide transportation to training facilities, and encourage participation in sport. These patterns suggest that single-parent households may shape a behavioral context in which adolescents have fewer opportunities for active engagement, increasing the likelihood of sedentary behaviors (2).

Screen exposure represents a related but distinct behavioral domain. In a cross-sectional Norwegian Health Behavior in School-aged Children (HBSC) sample, Langøy et al. (2) observed that adolescents residing in single-parent or reconstituted families reported greater weekday screen time compared to peers from dual-parent families. In parallel, Sisson et al. (14) reported that children from single-mother households were 49% more likely to have a bedroom television and 28% more likely to experience infrequent family meals, behaviors that may indirectly promote sedentary routines and suboptimal dietary habits. Although the absolute impact on weight gain is mediated by multiple factors, these findings collectively indicate that family structure can influence behavioral patterns that predispose adolescents to nutritional vulnerability (14).

However, the relationship between single-parent households and screen time is not uniform across populations. Using a nationally representative cross-sectional Canadian HBSC sample, McMillan et al. (3) reported that youth from single-parent and reconstituted families did not demonstrate significantly higher screen time than peers from traditional dual-parent households. These findings suggest that contextual variables, including socioeconomic status, access to supervised after-school activities, and cultural norms, may moderate the effects of family structure on sedentary behaviors. Such evidence highlights the importance of interpreting single-parent status as a context that can increase vulnerability under certain conditions, rather than as a universal predictor of poor behavioral or metabolic outcomes (3).

Meal patterns provide a separate domain of direct evidence concerning nutritional routines in single-parent households. Family meals have been repeatedly associated with higher intake of fruits and vegetables and lower consumption of energy-dense foods. Sisson et al. (14) and Yelick (15) reported that adolescents from single-parent households had fewer shared meals and less healthy dietary consumption patterns, characterized by higher intake of convenience and fast foods and reduced engagement in physical activity. Time pressures, financial limitations, and household instability were proposed as explanatory factors, as these constrain opportunities for meal preparation and limit parental involvement in structuring daily routines. In essence, single-parent households may provide a social and environmental context that increases the likelihood of behaviors associated with higher metabolic risk (14, 15).

Overall, direct evidence is limited-to-moderate for differences in family-meal frequency, dietary patterns, screen access or duration, and participation in structured physical activity across household structures, but remains limited for causal or metabolic inference. Findings vary across populations and socioeconomic settings, and the available studies do not identify which specific household characteristics account for these differences. Evidence directly linking single-parent context to loneliness, problematic digital media use, sleep disruption, emotional eating, or metabolic biomarkers also remains limited.

## 4 Loneliness and eating behavior

Loneliness in adolescence should be considered more than a transient emotional state. Except where family-structure analyses are explicitly identified, the available evidence is derived from general adolescent samples and should not be interpreted as direct evidence that the association between loneliness and eating behavior is stronger in single-parent households. It represents a psychosocial exposure that may shape eating behavior through stress reactivity, impaired emotion regulation, altered reward sensitivity, and reduced engagement in health-promoting routines. Adolescents who feel socially disconnected may be more vulnerable to using food as a compensatory strategy, particularly when negative emotions are persistent and alternative sources of emotional support are limited. An approximately 11-year prospective analysis from the HUNT Study followed participants from adolescence, at ages 13–19 years in 2006–2008, to young adulthood, at ages 23–31 years in 2017–2019. Frequent loneliness during adolescence was associated with a higher likelihood of BMI-defined obesity in young adulthood, while associations with waist circumference were partly sex-specific after covariate adjustment (16). The long follow-up and objectively measured anthropometric outcomes provide moderate temporal evidence that loneliness precedes later adiposity; however, the two-wave observational design, single-item assessment of loneliness, and possibility of residual confounding preclude causal inference.

Evidence from adolescent-parent dyads further supports the idea that loneliness may influence weight-related behavior within a family context. Mason reported that greater adolescent loneliness was positively associated with emotional eating and BMI-z, while parental loneliness was also associated with adolescent emotional eating. These findings are particularly relevant to the present review because they suggest that loneliness is not only an individual psychological state, but also a relational experience embedded within family dynamics (17, 18).

A four-wave Norwegian study followed participants from early adolescence to adulthood over 13 years, with an approximately two-year interval between the first two adolescent assessments. Using a random-intercept cross-lagged panel model, Cortés-García et al. (19) found that the temporal relationships between loneliness and disordered eating varied according to sex, developmental stage, and eating-behavior phenotype. In boys, loneliness during early-to-middle adolescence was associated with greater overall disordered eating and dieting approximately 2 years later, whereas bidirectional associations with bulimic symptoms were more evident among females during emerging adulthood. The four-wave design and separation of within-person from between-person effects provide comparatively stronger temporal evidence than conventional two-wave cohort analyses. Nevertheless, the observational design, attrition across 13 years, and developmental heterogeneity prevent causal interpretation (19).

These findings support a cautious but biologically and psychologically plausible interpretation: loneliness may increase vulnerability to maladaptive eating when it co-occurs with negative affect, poor self-regulation, or social withdrawal. Within the interpersonal model of eating disorders, socially disconnected individuals may use overeating, binge-like eating, or restrictive eating as maladaptive attempts to regulate emotions, distract from distress, or regain a sense of control. Conversely, disordered eating may itself promote shame, concealment, and avoidance of social situations, thereby reinforcing loneliness over time (20, 21).

Stress-related processes may help explain the observed association between loneliness and emotional eating, although mediation has not been established in adolescents. Adolescents often experience stress in multiple domains, including peer pressure, family strain, academic demands, financial concerns, and body-related worries. While acute stress may transiently suppress appetite in some individuals, chronic or repeated stress can increase food cravings, particularly for highly palatable, energy-dense foods. In this context, emotional eating can be understood as eating in response to negative emotions rather than physiological hunger (22–24). The PLACE-19 study provides useful adolescent-specific evidence for this pathway. In Polish female adolescents, excessive body weight was associated with higher stress in selected domains and higher emotional eating scores. Mediation analysis suggested that emotional eating partly explained the relationship between stress and body mass, although the direct pathway remained stronger. These findings indicate that stress-related eating should not be treated as the sole explanation for weight gain, but as one component within a broader network of psychological, behavioral, and environmental influences (25).

Emotion-regulation difficulties may modify the association between perceived stress and emotional eating. Spinner et al. (26) showed that the association between perceived stress and emotional eating was strongest among adolescents with higher emotion dysregulation, whereas stress was not significantly associated with emotional eating among adolescents with average or below-average dysregulation. This pattern suggests that emotion-regulation capacity may help explain heterogeneity in adolescents’ eating responses to perceived stress (26, 27). A four-year longitudinal study followed adolescents from age 15 to age 19 and assessed emotion regulation at age 15, eating behaviors at age 16, and objectively measured adiposity at age 19. Shriver et al. (28) found that poorer emotion regulation was indirectly associated with later adiposity through greater emotional eating, although emotion regulation showed no direct association with adiposity at follow-up. This study provides limited-to-moderate temporal evidence for an indirect pathway because of its small sample size (*N* = 153), observational mediation model, and absence of repeated adiposity assessment across all waves. Cross-sectional adolescent studies also associate emotional eating with adiposity and cardiometabolic correlates. In a Peruvian sample, emotional eating was positively associated with BMI, whereas mental well-being showed an inverse association with BMI (29). Ramírez-Garza et al. (30) further reported associations among emotional eating, adiposity, metabolic-syndrome indicators, dietary factors, and urinary 8-isoprostane. These findings indicate that emotional eating may coexist with broader metabolic vulnerability, but they do not establish its temporal or mediating role (30).

Sleep difficulties may co-occur with greater emotional reactivity and emotion-driven eating. Greater insomnia severity and shorter time in bed have been associated with emotional eating and body-dissatisfaction-related eating responses in adolescents, although the cross-sectional evidence cannot determine directionality (31). Cortisol dysregulation, reward processing, and appetite-related hormones provide plausible biological explanations, but direct adolescent evidence remains limited and heterogeneous. These mechanisms should therefore be interpreted as indirect biological support rather than as established mediators between loneliness and eating behavior (32–34).

Overall, adolescent loneliness is associated with emotional and stress-related eating, although the magnitude and direction of these relationships vary according to emotion regulation, sleep, sex, and developmental stage. Evidence concerning cortisol, appetite hormones, and reward signaling remains indirect. Across the longitudinal studies, follow-up ranged from approximately two to 13 years. Multiwave analyses strengthen temporal ordering, but the heterogeneity of outcomes, sex- and stage-specific findings, and observational designs indicate that the overall evidence remains moderate rather than causal. Loneliness is therefore best viewed as a psychosocial exposure that may interact with behavioral and family factors to shape eating patterns rather than as an independent metabolic mechanism.

## 5 Digital media and dietary behavior

Accordingly, the following synthesis distinguishes duration-based screen exposure from problematic or compulsive digital media use and evaluates their dietary associations in relation to duration, timing, content, platform, and impaired control (35, 36). Most studies in the available evidence base did not stratify their findings by family structure; therefore, their applicability to single-parent households remains indirect and should be interpreted in relation to household-level contextual modifiers. Recent evidence suggests that problematic forms of digital engagement may be more strongly related to unhealthy dietary behavior than high-frequency but non-problematic use. In a large cross-national analysis of 222,865 adolescents from 41 countries, problematic social media use was associated with lower odds of good dietary intake, lower daily fruit and vegetable consumption, reduced breakfast intake, and higher daily consumption of sweets and sugary drinks. Excessive social media use also showed unfavorable associations with some dietary indicators, although its pattern was more mixed, suggesting that addiction-like symptoms such as loss of control, preoccupation, and neglect of other activities may be more consistently associated with unfavorable dietary patterns than intensity of use alone (37).

Disruption of daily eating structure is one behavioral correlate repeatedly reported alongside problematic digital media use. Adolescents with prolonged online engagement may also report delayed or skipped meals, particularly breakfast; however, the available evidence does not establish whether digital use precedes these routines or reflects an already unstructured daily pattern. Khan et al. (37) reported that problematic social media use was associated with substantially lower odds of daily breakfast intake in both boys and girls, while studies summarized in the same article also linked screen-based behaviors such as general internet use, gaming, and television viewing to breakfast omission. These findings support an association between problematic digital use and less structured eating routines, without establishing the direction of that relationship (36, 38, 39). Late-night digital engagement may also coincide with delayed morning routines and breakfast omission; associations involving bedtime delay, sleep duration, and sleep quality are treated as a distinct sleep-related pathway. Snacking during screen use is another behavior reported alongside digital exposure, although total energy intake is rarely measured directly. Screen-based activities have been associated with distracted or mindless eating, in which adolescents consume snacks while scrolling, gaming, or watching videos with limited awareness of hunger, satiety, or portion size. Khan et al. (37) noted that adolescents with problematic social media use may consume unhealthy snacks while scrolling and may have less time or motivation for preparing structured meals, thereby increasing reliance on processed and convenient food options. Similarly, broader screen-time evidence suggests that television, video games, and personal computer use can increase motivated responses to food and snacking behavior in children and adolescents (37).

Fast food and sugar-sweetened beverages represent particularly relevant outcomes in this pathway. In a nationally representative Korean adolescent sample, longer smartphone screen time was associated with more frequent fast food intake, with a graded pattern across increasing screen-time categories. Adolescents using smartphones for six or more hours per day had a higher probability of frequent fast food consumption than those using smartphones for less than 2 h per day, and the association differed by content type, with user-generated video content among boys and social networking among girls showing notable associations with fast food intake (40). Exposure to eating-related entertainment content may intensify these associations. Eating shows, including mukbang and cookbang content, often normalize large portions, rapid intake, and highly palatable foods, while presenting eating as entertainment and social engagement. Joo et al. found that Korean adolescents exposed to eating shows were more likely to consume fast food, sugar-sweetened beverages, and high-caffeine drinks. Although such studies remain observational, they highlight the need to consider the qualitative nature of digital content, not only total screen time, when evaluating adolescent dietary risk (41).

Digital food marketing represents an additional exposure relevant to adolescent food choice. Unlike traditional advertising, digital marketing can be personalized, embedded within entertainment, and communicated through peers, influencers, or platform algorithms. Greater social-media use and recall of digital food marketing have been associated with more frequent consumption of ultra-processed foods and fast food, whereas associations with fruit and vegetable intake have been weaker or inconsistent (42). Qualitative findings further suggest that adolescents may not consistently recognize influencer- or peer-generated food content as advertising, allowing commercial messages to be interpreted as authentic recommendations or social trends. Such exposure may normalize branded snacks, desserts, sugary drinks, and fast foods, although the observational evidence does not establish that marketing exposure directly changes consumption (43).

Problematic internet use may also intersect with disordered and reward-driven eating. Aydin et al. (44) reported that problematic internet use, social media addiction, diet quality, and disordered eating risk were interconnected in adolescents, with internet addiction emerging as a central node in network analysis. Their mediation findings suggested that diet quality may partly explain the relationship between problematic internet use and disordered eating risk, although the effect size was modest and the cross-sectional design precluded causal inference. These findings support a network-based interpretation in which digital overuse, poor dietary patterns, body-related concerns, and disordered eating tendencies may reinforce one another rather than operate through a single linear pathway (44).

Prospective evidence has also examined whether screen exposure precedes changes in overall diet quality. Using data from the Adolescent Brain Cognitive Development (ABCD) Study, Nagata et al. (45) examined both cross-sectional associations at the Year 1 assessment and prospective associations from baseline to the one-year follow-up. Greater baseline total screen time and selected screen modalities were associated with lower nutrition scores 1 year later, whereas some modality-specific associations were observed only cross-sectionally. The prospective component provides limited temporal evidence because of the short follow-up, self-reported screen exposure, relatively small effect sizes, and the possibility of residual confounding. Their findings extend older television-focused research by showing that newer forms of screen exposure may have stronger or distinct nutritional correlates. These associations may reflect exposure to unhealthy food practices and advertising, increased snacking during screen use, and displacement of structured meals or food preparation (45). Collectively, duration-based screen exposure and problematic digital media use show overlapping but non-equivalent associations with adolescent dietary behavior. Heterogeneity in definitions and measurement instruments limits direct comparison, and findings should not be generalized from one construct to the other. Because the available prospective nutrition evidence is limited mainly to a one-year follow-up, temporal inference for the relationship between digital media exposure and later diet quality remains limited. Overall, the strength of evidence is therefore limited-to-moderate for dietary associations and limited for directionality or causality.

## 6 Sleep disruption and energy-related correlates

Sleep disruption is examined here in relation to late-night digital engagement, irregular eating, and weight-related behaviors. The relevant exposures differ from the direct dietary correlates of digital media and include nighttime device use, difficulty disengaging, bedtime delay, and sleep loss rather than digital content itself. Adolescents may be particularly susceptible because biological phase delay, school demands, and increasing autonomy already place sleep timing under pressure (46–48). The available evidence is derived predominantly from general adolescent samples, and direct evidence that single-parent household characteristics modify these associations remains limited. Problematic smartphone use has been consistently associated with poorer sleep quality in adolescents. In a Turkish adolescent sample, Bozkurt et al. (49) reported that nearly half of participants showed signs of problematic smartphone use, while the majority used smartphones in bed, often for social networking, video watching, messaging, and gaming. Poor sleep quality was associated with bedtime smartphone duration, the number of in-bed smartphone activities, problematic smartphone use, and bedtime procrastination. These findings support an association between nighttime digital engagement and the organization of sleep, but they do not establish that problematic smartphone use causes poor sleep or subsequent dietary change (49).

Bedtime procrastination describes delaying sleep without an external necessity and may help explain how digital engagement is associated with sleep restriction. Stimulating content, social interaction, fear of missing out, and loss of time awareness may make disengagement difficult. Cross-sectional mediation and qualitative studies, some conducted in young-adult rather than adolescent populations, suggest that bedtime procrastination may partly account for the association between problematic smartphone use and poor sleep quality; however, simultaneous or non-longitudinal measurement prevents temporal interpretation (50–53). Consistent associations with sleep latency, nighttime awakening, daytime sleepiness, and poor sleep quality have also been reported in adolescent samples, although these findings remain observational (54).

Psychological strain and pre-sleep arousal may further contribute to the association between problematic digital use and sleep. In Chinese adolescents, anxiety statistically mediated the relationship between problematic internet use and sleep quality, but the cross-sectional design does not demonstrate a sequential process (55).

Pre-sleep light exposure and cognitive arousal provide a separate mechanistic explanation for the association between evening digital use and sleep timing. Evening screen light, interactive content, and sustained social engagement may also influence melatonin-related timing and cognitive arousal. Experimental and mechanistic evidence supports the plausibility of these effects, but does not establish the long-term consequences of habitual digital use in adolescents (56–58).

Short sleep has been examined in relation to appetite-related hormones and eating behavior, but the findings remain inconsistent. A recent systematic review reported heterogeneous and frequently non-significant changes in leptin and ghrelin following sleep deprivation, precluding their interpretation as established mediators in adolescents (59). Short sleep has also been associated with negative affect, inhibitory control, reward responsiveness, snacking, and food preference, although much of this evidence is indirect or derived from populations outside the specific focus of this review (60–62). These findings do not establish that digital media use causes appetite dysregulation through sleep loss. In a cross-sectional Chinese adolescent sample, insufficient sleep and high screen-based sedentary time were independently associated with overweight, and their co-occurrence was associated with the highest odds of overweight. These findings support behavioral clustering rather than an additive causal effect, particularly because energy intake, expenditure, and net energy balance were not directly measured. The proposed explanations involving appetite hormones, fatigue, physical activity, snacking, and food advertising should therefore be considered hypotheses requiring prospective testing (63).

Two prospective ABCD analyses provide moderate temporal evidence over approximately 2 years. Among adolescents with overweight or obesity, shorter sleep duration was associated with a lower likelihood of transitioning to a normal BMI percentile over a median follow-up of 657 days, whereas screen time was not associated with this transition (64). In a separate two-year analysis, higher screen time and insufficient sleep were independently and jointly associated with higher BMI and waist circumference at follow-up (65). These studies establish that the behavioral exposures preceded the measured weight outcomes, but they do not demonstrate causality because sleep and screen exposure were observationally measured, some measures were parent- or self-reported, and residual confounding remains possible. Evidence that problematic social media use co-occurs with poorer sleep and disordered eating supports overlap among these behaviors, but does not establish that sleep mediates the association between digital use and eating. Heterogeneity in exposure definitions and outcome measures further limits pathway-level inference (5).

Current evidence associates late-night or problematic digital engagement with delayed bedtime, shorter sleep duration, and poorer sleep quality. Sleep disruption is also associated with irregular eating, fatigue, and reduced activity, although these relationships remain potentially bidirectional. Prospective findings provide limited-to-moderate temporal support for later weight-related outcomes but do not establish a causal sequence from digital media use through sleep disruption to metabolic risk. Table 2 summarizes the distinct dietary, sedentary, and sleep-related pathways and the evidential caution required for their interpretation.

**Table 2:** Evidence map of psychosocial, digital, eating-related, sedentary, and sleep-related associations in adolescents.

Domain	Main exposure	Associated behavioral process	Nutritional/eating outcome	Possible downstream correlate	Evidence interpretation	Evidence basis for the single-parent context
Psychosocial distress	Loneliness/perceived social isolation	Negative affect, emotion dysregulation, stress-related coping	Emotional eating, stress eating, preference for energy-dense foods	Possible association with greater energy intake or poorer dietary control; rarely measured directly	Predominantly observational; directionality may be bidirectional	*Indirect*: evidence is derived predominantly from general adolescent samples; stronger or modified associations in single-parent households have not been established.
Family context	Single-parent household constraints	Reduced meal structure, limited supervision, fewer shared meals	Irregular meals, convenience food reliance, reduced fruit/vegetable intake	Less structured eating and activity routines	Contextual vulnerability, not deterministic causality	*Direct and mixed*: Single-parent studies support differences in family meals, dietary routines, and some weight-related behaviors, but the complete behavioral pathway has not been directly tested.
Digital exposure	Problematic internet/social media use	Displacement of structured routines and meals	Breakfast skipping, delayed meals, meal irregularity	Reduced dietary structure; later compensatory intake remains insufficiently tested	Mostly observational evidence	*Indirect*: Most studies do not stratify results by family structure; applicability to single-parent households is based on contextual extrapolation.
Screen-based eating	Smartphone, gaming, video streaming	Distracted eating, reduced satiety awareness, mindless snacking	Increased snacking, larger portions, ultra-processed snacks	Concurrent snacking or larger portions reported in some studies	Mostly cross-sectional; total energy intake rarely measured	*Indirect*: Evidence is derived mainly from broader adolescent populations, with little direct assessment in single-parent samples.
Digital food environment	Social media food marketing, influencers, eating shows	Food cue exposure, peer norms, reward-based cravings	Fast food, sweets, sugar-sweetened beverages, high-caffeine drinks	Greater reported intake of palatable, energy-dense products	Content and context of screen use are important	*Indirect*: Family-structure-specific evidence is lacking; the proposed relevance to single-parent households remains contextual.
Sedentary behavior	Prolonged screen time	Reduced physical activity and prolonged sitting	Lower energy expenditure; possible concurrent snacking	Lower activity and possible concurrent snacking; net energy balance rarely measured	Mixed observational evidence	*Mixed*: Some single-parent studies report differences in screen exposure or participation in organized physical activity, whereas the broader energy-balance pathway is supported mainly by general adolescent evidence.
Sleep disruption	Bedtime procrastination / short sleep	Appetite dysregulation, fatigue, reward sensitivity	Cravings, emotional eating, lower dietary control	Association with later weight-related outcomes in some prospective studies	Limited-to-moderate temporal evidence; causality not established	*Indirect*: Sleep and appetite associations are supported primarily by general adolescent studies; modification by single-parent household characteristics remains untested.

Direct evidence refers to studies that explicitly analyzed adolescents from single-parent or single-mother households. Indirect evidence refers to findings from broader adolescent populations that are used to assess contextual plausibility but do not establish stronger or different associations in single-parent households. Mixed evidence indicates that some components are supported by single-parent studies, whereas the proposed pathway as a whole relies partly on extrapolation.

## 7 Household characteristics as contextual modifiers

Direct studies document differences in adolescent routines across household structures but rarely test whether specific household characteristics modify the associations among loneliness, digital media use, sleep, and eating behavior. The role of single-parent context in these broader relationships therefore remains inferential rather than empirically established. The relevant question is not whether single-parent status itself causes adverse outcomes, but whether particular household conditions alter psychosocial exposure, coping opportunities, and the organization of daily routines (1, 2, 14, 15). Candidate contextual modifiers include parental work schedules, available time, economic resources, evening supervision, household stability, task distribution, access to structured activities, and social support. Broader adolescent family-assistance studies suggest that young people may contribute through household chores, shopping, meal preparation, or sibling care when family needs increase (66, 67). However, these findings are not specific to single-parent households. Where such responsibilities are substantial, they could constrain time available for regular meals, physical activity, or sleep, but this possibility requires direct assessment rather than assumption. Digital media may likewise provide social connection or emotional coping in some circumstances while facilitating prolonged or late-night engagement in others.

The relevance of these relationships is therefore likely to depend on the distribution of time, responsibilities, resources, supervision, and support rather than on family structure alone (68). These conditions may intensify or buffer the associations among loneliness, problematic digital media use, eating behavior, and sleep, and their effects may vary across socioeconomic and cultural settings. Single-parent status should therefore be treated as a heterogeneous contextual marker rather than an independent determinant of nutritional or metabolic outcomes. Figure 1 summarizes these household-level conditions as candidate contextual modifiers within a conceptual framework of partially overlapping associations rather than a verified temporal or causal sequence.

**Figure 1:**
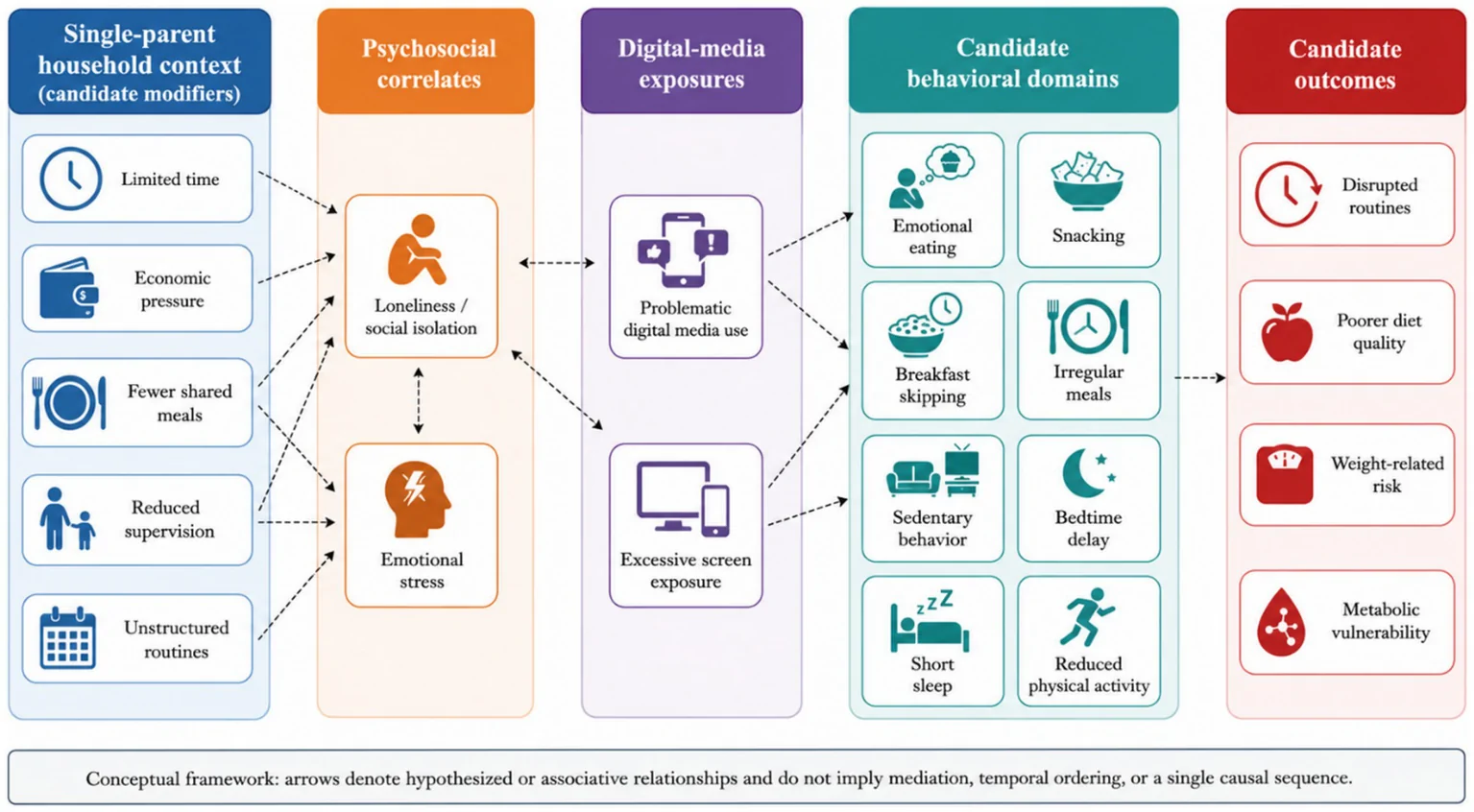
Conceptual framework summarizing candidate and partially overlapping associations among single-parent household context, psychosocial correlates, digital-media exposures, behavioral domains, and nutritional or metabolic outcomes in adolescents. Arrows indicate hypothesized or associative relationships and do not imply mediation, temporal ordering, or a single causal sequence.

## 8 Metabolic implications and evidential limits

Direct biomarker evidence linking loneliness or problematic digital media use to metabolic outcomes in single-parent adolescents is scarce. Consequently, the metabolic interpretation relies primarily on indirect evidence from broader adolescent research on sleep, stress, obesity, and metabolic regulation. A useful starting point is the observation that problematic internet use has been associated with overweight and obesity in adolescent populations. Abera et al. (69) found that different levels of problematic internet use were associated with higher odds of overweight/obesity after adjustment for confounders. Skipping breakfast, low physical activity, and prolonged sedentary time were also independently associated with weight status, indicating that problematic internet use may coexist with a broader cluster of behavioral characteristics. However, these findings do not demonstrate that digital use produces positive energy balance or independently causes adiposity (69). Metabolic syndrome in young populations is generally characterized by a cluster of cardiometabolic abnormalities, including central obesity, dyslipidemia, elevated blood pressure, hyperglycemia, and insulin resistance. Although diagnostic criteria in children and adolescents remain inconsistent, most definitions recognize abdominal adiposity and insulin resistance as important components (70–72). Established obesity research supports associations between excess adiposity and impaired insulin sensitivity through ectopic lipid accumulation, inflammation, oxidative stress, and altered adipokine signaling. These mechanisms describe downstream obesity-related biology but do not establish that loneliness, problematic digital media use, or single-parent household characteristics initiate this progression (73).

Sleep and circadian evidence provides a more direct metabolic anchor. In adolescents with overweight or obesity, shorter sleep duration, later bedtime, and later circadian timing were associated with lower insulin sensitivity, although the small observational sample limits generalizability (74). Broader evidence suggests that sleep loss and circadian misalignment may coincide with altered glucose regulation through neuroendocrine, autonomic, inflammatory, dietary, and activity-related processes. These findings support sleep disruption as a potential contributor, rather than a sufficient cause, of impaired insulin sensitivity (75, 76). Appetite-related hormones remain candidate rather than established mediators. Although some experimental studies have reported higher ghrelin and lower leptin after sleep restriction, a recent systematic review found no consistent changes and substantial heterogeneity across sampling protocols, participant characteristics, and study designs (59). Ghrelin may also participate in mood, stress, and circadian regulation, but limited adolescent evidence and possible isoform-specific effects preclude its interpretation as a demonstrated biomarker pathway in the target population (77).

Stress biology provides another plausible but indirect route. Chronic activation of the hypothalamic–pituitary–adrenal axis may influence appetite, adipose distribution, and metabolic regulation, yet cortisol responses vary across individuals and contexts and cannot provide a simple explanation for obesity (78). Pediatric obesity studies have associated endocrine stress markers or ostracism-related cortisol reactivity with food addiction, body composition, metabolic phenotype, or motivation for high-calorie foods. However, these studies did not examine the combined effects of single-parent household characteristics, loneliness, and problematic digital media use (79, 80).

Reward-related processes may likewise contribute to eating vulnerability during adolescence. Emotional distress, sleep loss, and exposure to online food cues may coincide with stronger cue-driven motivation and weaker inhibitory control. Neuroimaging and food-cue studies have associated overweight and obesity with altered responses to palatable food, but these findings should not be generalized to all adolescents or interpreted as evidence that digital media use directly alters dopamine signaling (81). Reviews of binge eating, food addiction, and adolescent brain development support the relevance of reward and inhibitory-control systems, while direct evidence for the proposed combined pathway remains absent (82–84).

Overall, the available evidence does not support a continuous sequence from household context or psychosocial exposure to metabolic dysfunction. Separate bodies of evidence associate problematic digital media use with sedentary behavior, meal irregularity, and sleep disruption; loneliness with emotional eating; and sleep or circadian measures with appetite-related and insulin-related outcomes in selected adolescent studies. Whether these associations combine to influence adiposity, insulin resistance, or metabolic syndrome has not been directly tested, particularly in adolescents from single-parent households. Figure 2 summarizes candidate and partially overlapping associations rather than verified intermediate mechanisms. Given the predominantly observational and indirect nature of the available evidence, these connections should be interpreted as a conceptual synthesis rather than a temporally established causal sequence.

**Figure 2:**
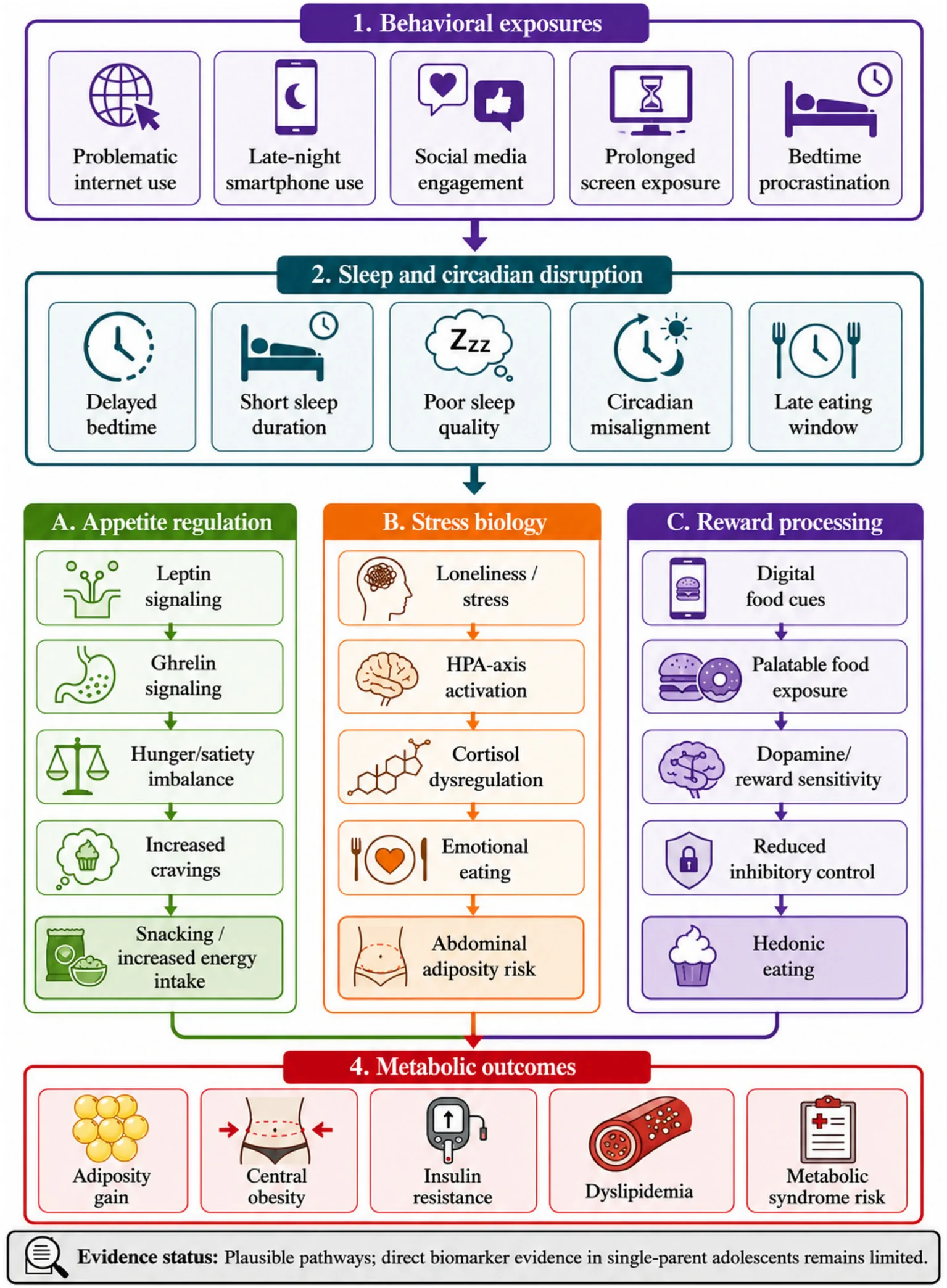
Evidence-calibrated conceptual map of associations among digital engagement, sleep and circadian factors, eating-related processes, and metabolic correlates. Arrows indicate hypothesized or associative relationships and do not imply temporal ordering, mediation, or causality.

## 9 Intervention priorities

Direct intervention evidence for adolescents from single-parent households remains limited; therefore, recommendations should be prioritized according to their proximity to the observed behavioral evidence, feasibility under household time and resource constraints, and potential for delivery without stigmatizing family structure. On this basis, the first priority is stabilization of daily eating and evening routines. A second priority is the provision of compensatory support through schools when household schedules or resources restrict access to meals and structured activities. More specialized psychosocial or digital-behavior interventions should be targeted to adolescents with identified loneliness, problematic digital media use, or persistent sleep difficulties rather than offered as universal components of every program. The highest-priority target is the establishment of predictable, low-burden routines around meals, evening digital use, and sleep. Practical strategies include reliable access to breakfast, planned after-school snacks, affordable and easily prepared meals, device-free eating, predefined evening screen limits, bedtime phone storage, and a consistent sleep schedule. These strategies address the most directly supported behavioral domains in the review, including fewer shared meals and less structured routines in some single-parent samples, as well as broader adolescent associations of problematic digital use with breakfast omission, irregular eating, and sleep disruption. Family-oriented guidance should therefore emphasize a small number of achievable routines rather than comprehensive lifestyle modification. Evidence from family-based interventions supports parental involvement, behavioral goal setting, and home-based reinforcement, although effects vary across settings and long-term metabolic outcomes remain uncertain (85–88).

Schools represent the second priority because they can provide predictable support without relying entirely on parental time, transportation, or financial resources. Universal or easily accessible school breakfast programs may improve breakfast participation and reduce the practical consequences of irregular morning schedules, although evidence for effects on cognition or metabolic outcomes remains mixed (89). Schools can also provide structured opportunities for physical activity and reinforce practical guidance on digital use and sleep. These components should be integrated into existing health or wellbeing programs rather than delivered as numerous separate interventions. Available evidence suggests that multicomponent school programs may modestly improve screen-related or activity behaviors, but effects on BMI are inconsistent; proximal outcomes such as breakfast participation, sleep timing, screen use, and physical activity should therefore remain the primary evaluation targets (90–92).

Targeted psychosocial and digital-behavior support constitutes a third, conditional priority. Adolescents reporting persistent loneliness, emotional distress, loss of control over digital use, or substantial bedtime delay may benefit from counseling, peer-support or school-belonging initiatives, and structured training in digital self-monitoring, notification management, and disengagement from nighttime media. These interventions should not be justified solely by single-parent household status, because direct evidence that family structure modifies these associations is lacking. Instead, eligibility should be based on assessed behavioral or psychosocial need. Evidence for school-based e-health, media-literacy, and psychosocial interventions remains emerging, and these strategies should be considered adjunctive rather than primary nutritional or metabolic interventions (93, 94).

Accordingly, a coordinated school–family approach should not be interpreted as requiring every intervention component for every adolescent. A stepped model is more defensible: universal low-burden support for meal and evening routines; school-based access to breakfast and structured activity where needed; and targeted psychosocial or digital-behavior support for adolescents with identified difficulties. Routine metabolic screening, intensive clinical intervention, or biomarker monitoring cannot currently be recommended on the basis of family structure or digital behavior alone and should remain limited to clinical indications or research settings. Intervention studies should initially evaluate proximal and measurable outcomes, including breakfast participation, meal regularity, bedtime screen exposure, sleep timing, diet quality, and physical activity, before testing longer-term effects on adiposity or cardiometabolic markers. Table 3 summarizes the prioritized intervention targets, with emphasis on strategies that are feasible for schools and adaptable to the practical constraints of single-parent families.

**Table 3:** Prioritized intervention targets for adolescents from single-parent households.

Priority	Target domain	Practical intervention strategy	Primary setting	Expected short-term outcome	Longer-term outcome to test	Key research gap
Priority 1: core and broadly applicable	Family nutrition routines	Low-burden family guidance on simple meal planning, affordable nutrient-dense foods, healthier snack availability, and predictable meal timing	Home; school–family programs	More regular meals, healthier snack availability, and reduced reliance on convenience foods and sugar-sweetened beverages	Improved diet quality and greater stability of weight-related trajectories	Few interventions have been adapted to the time and resource constraints of single-parent households
Priority 1: core and broadly applicable	Breakfast access and morning routines	Universal school breakfast, breakfast-in-classroom models, and practical support for consistent morning eating	School	Greater breakfast participation and more consistent morning routines	Improved diet quality and school attendance; effects on adiposity remain uncertain	Limited evidence directly linking school breakfast interventions with longer-term nutritional or metabolic outcomes
Priority 1: core and broadly applicable	Evening digital and sleep routines	Device-free meals, predefined evening screen limits, bedtime phone storage, notification management, consistent bedtimes, and reduced pre-sleep screen exposure	Home with school reinforcement	Reduced bedtime screen exposure, more consistent sleep timing, and improved sleep-related routines	Improved sleep duration and quality; possible improvements in eating regularity and activity patterns	Direct evidence in adolescents from single-parent households is lacking, and long-term weight-related effects remain uncertain
Priority 2: compensatory school-based support	Physical activity and daily structure	Structured school activity, active breaks, accessible after-school programs, and routine-focused health education	School; community	Increased participation in physical activity and greater daily routine stability	Improved activity patterns and possible favorable weight-related outcomes	Access, participation, and intervention effects may differ according to household resources, transportation, and socioeconomic conditions
Priority 3: targeted according to assessed need	Problematic digital-media use	Digital self-monitoring, goal setting, media literacy, time-management strategies, notification control, and support for disengagement from nighttime media	School counseling; appropriate e-health or community services	Improved awareness and control of digital use and reduced interference with meals or sleep	Reduced problematic digital-media use and improved behavioral functioning	Objective smartphone-use measurement and targeted intervention studies remain limited
Priority 3: targeted according to assessed need	Loneliness and psychosocial distress	Counseling, peer mentoring, school-belonging initiatives, supervised social activities, and referral when clinically indicated	School; community; appropriate mental-health services	Reduced loneliness and emotional distress and improved perceived social support	Possible reductions in emotional eating and improvements in wellbeing	Few intervention studies jointly assess loneliness, eating behavior, sleep, and weight-related outcomes

Priority levels reflect the directness of the available evidence, feasibility under household time and resource constraints, and suitability for broad versus needs-based delivery. They do not represent formally graded clinical recommendations. Priority 1 identifies low-burden strategies suitable for broad implementation; Priority 2 identifies compensatory supports that can reduce dependence on household resources; and Priority 3 includes targeted interventions that should be offered according to assessed psychosocial or behavioral need rather than single-parent household status alone. Longer-term metabolic monitoring remains an important research outcome but is not presented as a stand-alone intervention target.

## 10 Evidence gaps and research priorities

Despite growing evidence across these domains, the literature remains fragmented, with most studies examining only a small subset of the relevant contextual, psychosocial, behavioral, nutritional, or metabolic factors. The principal gaps concern temporal ordering, biomarker integration, household-level effect modification, measurement quality, and intervention evidence.

A major gap is the limited number of longitudinal studies. As summarized in Supplementary Table S1, the available longitudinal studies vary considerably in follow-up duration, number of assessment waves, measurement quality, and capacity to establish temporal ordering. Much of the current evidence remains cross-sectional and therefore cannot establish temporal ordering or directionality. Future longitudinal studies should test reciprocal relationships among household context, loneliness, digital-media use, sleep, and eating behavior and determine whether changes in these domains precede adiposity or metabolic outcomes. Another important limitation is the scarcity of biomarker-informed studies. Most evidence relies on BMI and self-reported behavioral measures, with few studies assessing whether the reviewed psychosocial and behavioral factors correspond to changes in body composition, insulin-related outcomes, inflammatory markers, or endocrine regulation. Longitudinal biomarker-informed research is needed to determine whether behavioral clustering is accompanied by measurable metabolic change.

A separate major gap is the absence of studies that directly test whether household time constraints, task distribution, supervision, economic resources, and social support modify the associations among loneliness, digital media use, eating behavior, sleep, and metabolic outcomes. Future studies should include sufficiently powered family-structure analyses and measure these household characteristics directly rather than treating single-parent status as a homogeneous demographic category. Such work should identify both risk-amplifying and protective conditions while avoiding stigmatizing assumptions about family structure. Few studies assess the principal psychosocial, behavioral, nutritional, and metabolic domains within the same design. Such studies should compare reciprocal and moderating relationships rather than assume a single sequential pathway. Measurement quality is another major limitation. Self-reported screen use, dietary intake, physical activity, and sleep are vulnerable to recall and social-desirability biases. Future studies should combine validated self-report measures with actigraphy, smartphone-use logs, ecological momentary assessment, wearable activity monitoring, time-stamped food records, and digital phenotyping. These approaches would clarify whether late-night screen exposure, breakfast omission, snacking, sedentary periods, and sleep restriction cluster within the same individuals and change together over time.

Intervention studies are also urgently needed. Current evidence is stronger for associations than for prevention or treatment strategies. School-based and family-centered programs may reduce screen time, improve physical activity, support breakfast intake, and strengthen sleep routines, but long-term effects on metabolic outcomes remain uncertain. Future intervention studies should test a stepped model centered first on feasible meal and evening-routine support, with school-based access and targeted psychosocial or digital-behavior components added according to identified need. This approach would clarify whether a focused core intervention is sufficient or whether selected adolescents benefit from additional components. For single-parent families, these programs should be feasible, low-cost, flexible, and sensitive to time and resource constraints. Primary outcomes should include meal regularity, breakfast participation, bedtime screen exposure, sleep timing, diet quality, and physical activity. Adiposity and cardiometabolic indicators should be examined as longer-term exploratory outcomes until stronger intervention evidence becomes available. Greater attention should also be paid to equity and contextual adaptation. Adolescents from single-parent households are not a homogeneous group, and their experiences may vary by socioeconomic status, gender, culture, parental employment, neighborhood safety, digital access, and school support. Interventions designed for two-parent or resource-rich households may not translate well to families with limited time, unstable schedules, or restricted access to healthy foods and recreational opportunities. Future studies should therefore examine whether intervention effects differ across socioeconomic groups, family structures, and cultural contexts. Equity-oriented designs should avoid blaming families and instead focus on reducing barriers to healthy routines. Together, these priorities support developmentally informed and context-sensitive research that combines stronger temporal designs with improved behavioral and metabolic measurement. The main evidence gaps and methodological priorities are summarized in Table 4.

**Table 4:** Key research gaps and future directions for integrated studies on single-parent adolescents, digital behavior, nutrition, and metabolic risk.

Research gap	Why it matters	Recommended study design	Suggested variables/outcomes
Lack of longitudinal studies	Directionality is unclear	Prospective cohort studies	loneliness, problematic internet use, sleep, diet, and BMI trajectory
Limited biomarker data	Mechanisms remain speculative	Biomarker-rich observational studies	fasting insulin, homeostatic model assessment of insulin resistance (HOMA-IR), lipids, cortisol, leptin, ghrelin
Few studies on single-parent adolescents	Population-specific vulnerability is underexplored	Stratified or targeted adolescent studies	family structure, parental time, meal routines, supervision
Lack of integrated models	Current evidence is fragmented	structural equation modeling, mediation, moderation, network analysis	loneliness, problematic internet use, diet, sleep, and metabolic outcomes
Limited intervention trials	Evidence is mostly observational	Multicomponent RCTs	digital self-regulation, sleep support, breakfast routines, psychosocial support
Weak objective measurement	Self-report bias is common	Mixed-method and digital phenotyping studies	actigraphy, phone logs, food records, ecological momentary assessment

## 11 Conclusion

Adolescents in single-parent households may encounter time, financial, supervisory, and routine-related constraints that shape nutritional behavior, but family structure alone does not explain metabolic vulnerability. Across broader adolescent populations, loneliness is associated with emotional eating, while problematic digital media use is associated with irregular meals, distracted eating, poorer diet quality, and sleep disruption. These factors may cluster with sedentary behavior and adiposity, although their temporal order and metabolic consequences remain uncertain.

The current evidence is limited by its predominantly observational design and the scarcity of studies that directly examine single-parent adolescents using integrated behavioral and metabolic measures. Future research should combine longitudinal assessment of household context, psychosocial wellbeing, digital use, diet, sleep, and objective metabolic outcomes. Until such evidence is available, prevention should prioritize feasible meal routines, reduced late-night digital use, sleep support, and access to psychosocial resources rather than assume a uniform risk pathway across single-parent families.
