# Supplementary material for: Loneliness, problematic digital media use, and nutritional–metabolic vulnerability in adolescents from single-parent households: an integrative review of eating- and sleep-related pathways
Source: Front Nutr. 2026 Sep 22;13:1888958. doi: 10.3389/fnut.2026.1888958 (PMC13639669; doi:10.3389/fnut.2026.1888958)
Supplement: Supplementary file 1 [file Table_1.docx]

**Supplementary Table S1.** Longitudinal studies and strength of temporal inference

| **Domain** | **Follow-up** | **Longitudinal design** | **Temporal inference** | **Principal limitation** | **Ref.** |
| --- | --- | --- | --- | --- | --- |
| Family structure and obesity | Approximately 3 years, from 9 months to preschool age | Repeated family-structure history | Limited-to-moderate | Preschool population; observational exposure trajectories | [13] |
| Loneliness and adult adiposity | Approximately 11 years | Two-wave prospective cohort | Moderate | Single-item loneliness; residual confounding | [16] |
| Loneliness and disordered eating | 13 years; four waves | Random-intercept cross-lagged panel model with within-person analysis | Comparatively stronger | Attrition; developmental and sex heterogeneity; observational | [19] |
| Emotion regulation, eating, and adiposity | 4 years | Three-wave longitudinal pathway analysis | Limited-to-moderate | Small sample; indirect mediation model | [28] |
| Screen time and diet quality | 1 year | Prospective and cross-sectional ABCD analyses | Limited | Short follow-up; self-reported exposure; small effects | [45] |
| Screen time, sleep, BMI, and waist circumference | 2 years | Prospective ABCD analysis | Moderate | Observational exposures; residual confounding | [65] |
| Sleep, screen time, and BMI-category transition | Median 657 days | Prospective ABCD analysis | Moderate | Short follow-up; parent-reported sleep; screen-time result was null | [64] |

Temporal-inference grades indicate the relative capacity of each study to establish temporal ordering and should not be interpreted as evidence of causality. Byrne, Cook [12], Langøy, Smith [2], and McMillan, McIsaac [3] were not classified as longitudinal studies because the associations reported in these publications were based on cross-sectional analyses, despite the use of data from named cohort or surveillance datasets.

**Supplementary Table S2.** Qualitative appraisal of evidence strength across the principal review domains

| **Evidence domain** | **Predominant evidence designs** | **Directness to single-parent adolescents** | **Principal strengths** | **Principal limitations and risk-of-bias concerns** | **Overall qualitative strength** |
| --- | --- | --- | --- | --- | --- |
| Single-parent household context and behavioral outcomes | Systematic review, cross-sectional studies, and selected longitudinal family-structure analyses | Relatively direct for family meals, screen-related routines, physical activity, and weight-related outcomes | Direct analysis of family structure in several studies; some nationally representative samples; selected longitudinal family-structure histories | Predominantly observational evidence; inconsistent findings across settings; socioeconomic confounding; some evidence derived from children rather than adolescents | Limited-to-moderate for behavioral associations; limited for causal or metabolic inference |
| Loneliness and eating- or weight-related outcomes | Cross-sectional studies, parent–adolescent studies, two-wave cohorts, and multiwave longitudinal analyses | Mostly indirect because most studies did not stratify by family structure | Long follow-up in selected cohorts; one multiwave within-person analysis; consistency for associations with emotional or disordered eating | Self-reported exposures; residual confounding; attrition; heterogeneity by sex, developmental stage, and eating phenotype | Moderate for association and temporal ordering; limited for single-parent-specific and causal inference |
| Digital media use and dietary behavior | Large cross-sectional surveys, qualitative studies, network analyses, reviews, and one-year prospective evidence | Predominantly indirect | Large multinational samples; differentiation of screen duration, content, and problematic use; limited prospective support | Self-reported digital exposure and diet; reverse causation; small prospective effects; heterogeneous definitions; few direct single-parent analyses | Limited-to-moderate for dietary associations; limited for directionality and causality |
| Problematic digital use and sleep | Systematic reviews, cross-sectional and mediation studies, and selected experimental or mechanistic studies | Predominantly indirect | Relatively consistent associations with bedtime delay, sleep duration, and sleep quality; some experimental support for evening-light mechanisms | Predominantly observational evidence; self-reported sleep or digital use; cross-sectional mediation; inclusion of some young-adult samples; heterogeneous exposure definitions | Moderate for association; limited for mediation and causal sequencing |
| Sleep, circadian factors, adiposity, and metabolic outcomes | Prospective cohorts, small observational biomarker studies, systematic reviews, and meta-analyses | Indirect for the target household context | Prospective evidence for selected weight-related outcomes; objective sleep or metabolic measures in some studies | Residual confounding; small biomarker samples; inconsistent leptin and ghrelin findings; limited direct measurement of energy balance | Limited-to-moderate for weight-related and metabolic associations; limited for biological mediation |
| Stress, appetite hormones, and reward-related mechanisms | Mechanistic reviews, pediatric obesity studies, experimental studies, and neuroimaging evidence | Largely indirect | Provides biological plausibility and identifies candidate mechanisms | Target exposures were rarely measured together; several studies involved clinical, adult, or obesity-selected samples; substantial indirectness | Limited and hypothesis-generating |
| Intervention evidence | Systematic reviews, meta-analyses, completed interventions, and trial protocols | Very limited for single-parent adolescents | Some evidence for proximal changes in screen use, sleep routines, physical activity, and family-based behaviors | Intervention heterogeneity; short follow-up; inconsistent effects on BMI; few target-population trials; limited metabolic outcomes; protocols provide no effectiveness data | Limited-to-moderate for proximal behavioral outcomes; limited for single-parent-specific and metabolic outcomes |

**Note:** Qualitative ratings reflect confidence in the specific inference addressed within each domain and should not be interpreted as a universal ranking based solely on study design. Systematic reviews and meta-analyses were evaluated according to the quality and design of their included studies. The ratings do not establish causality and were not used as formal exclusion criteria.
